# Supplementary material for: Kinetic sculpting of the seven stripes of the Drosophila even-skipped gene
Source: eLife. 2020 Dec 10;9:e61635. doi: 10.7554/eLife.61635 (PMC7864633; doi:10.7554/eLife.61635)
Supplement: Supplementary file 1. [file elife-61635-supp1.zip › Berrocal_2020_File_S1.tgz!/Berrocal_2020/Figures/Figure8.pdf]

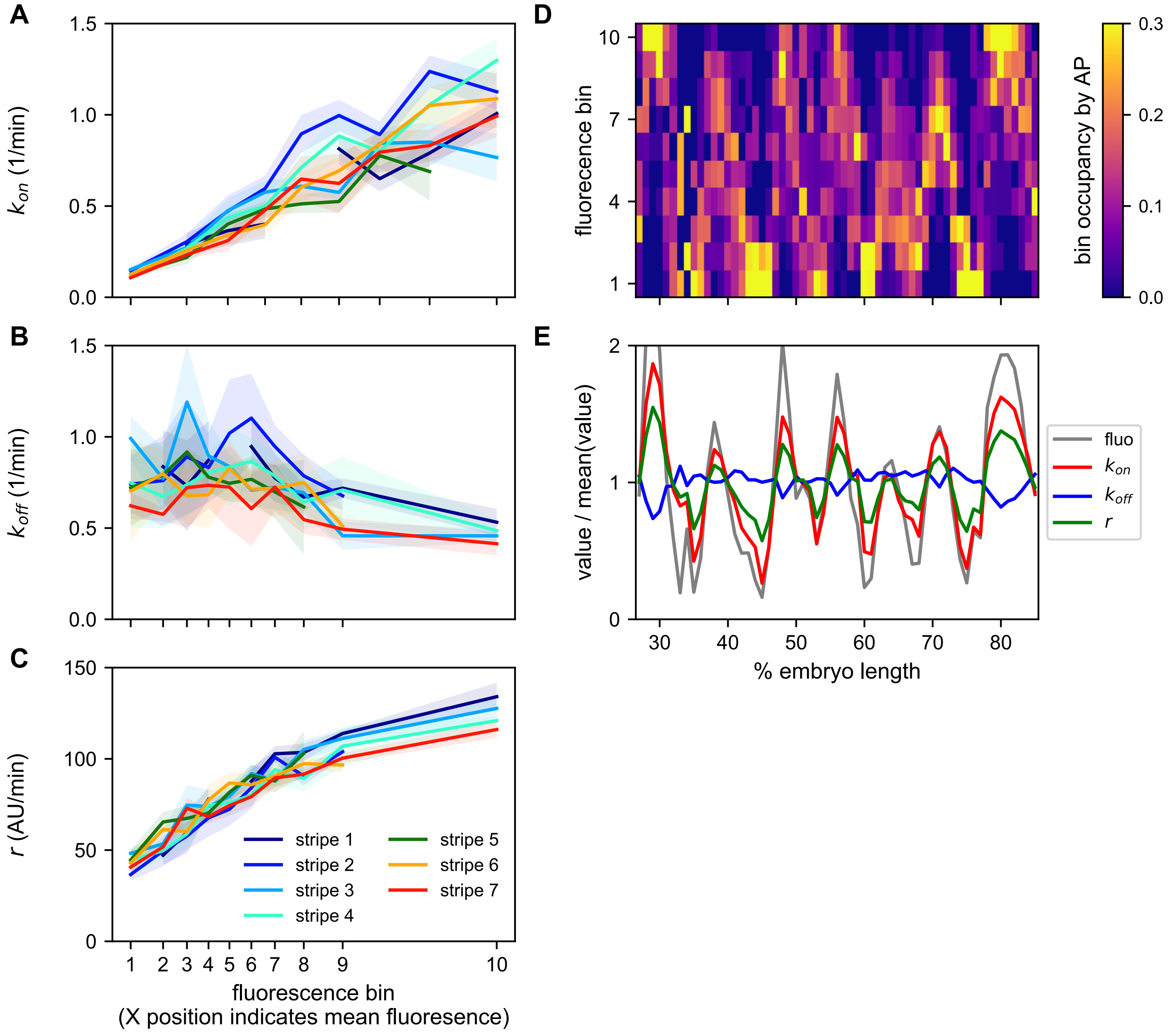

Figure 8. A common bursting control mechanism across all even-skipped stripes. (A-C) cpHMM inference was carried over nuclei binned according to their average fluorescence value indicating that while (A) kon and (C) r are subject to the same regulation along all stripes, (B) koff remains unchanged. Error bars are calculated by taking the standard deviation across cpHMM inference results for multiple bootstrapped samples of experimental data. (D) Distribution of average nuclear fluorescence values along the AP axis. (E) Mean nuclear fluorescence values for each AP position together with the corresponding averaged and weighted bursting parameters.rsting control mechanism across all even-skipped stripes.
