# Supplementary material for: Kinetic sculpting of the seven stripes of the Drosophila even-skipped gene
Source: eLife. 2020 Dec 10;9:e61635. doi: 10.7554/eLife.61635 (PMC7864633; doi:10.7554/eLife.61635)
Supplement: Supplementary file 1. [file elife-61635-supp1.zip › Berrocal_2020_File_S1.tgz!/Berrocal_2020/Figures/Figure7-S1.pdf]

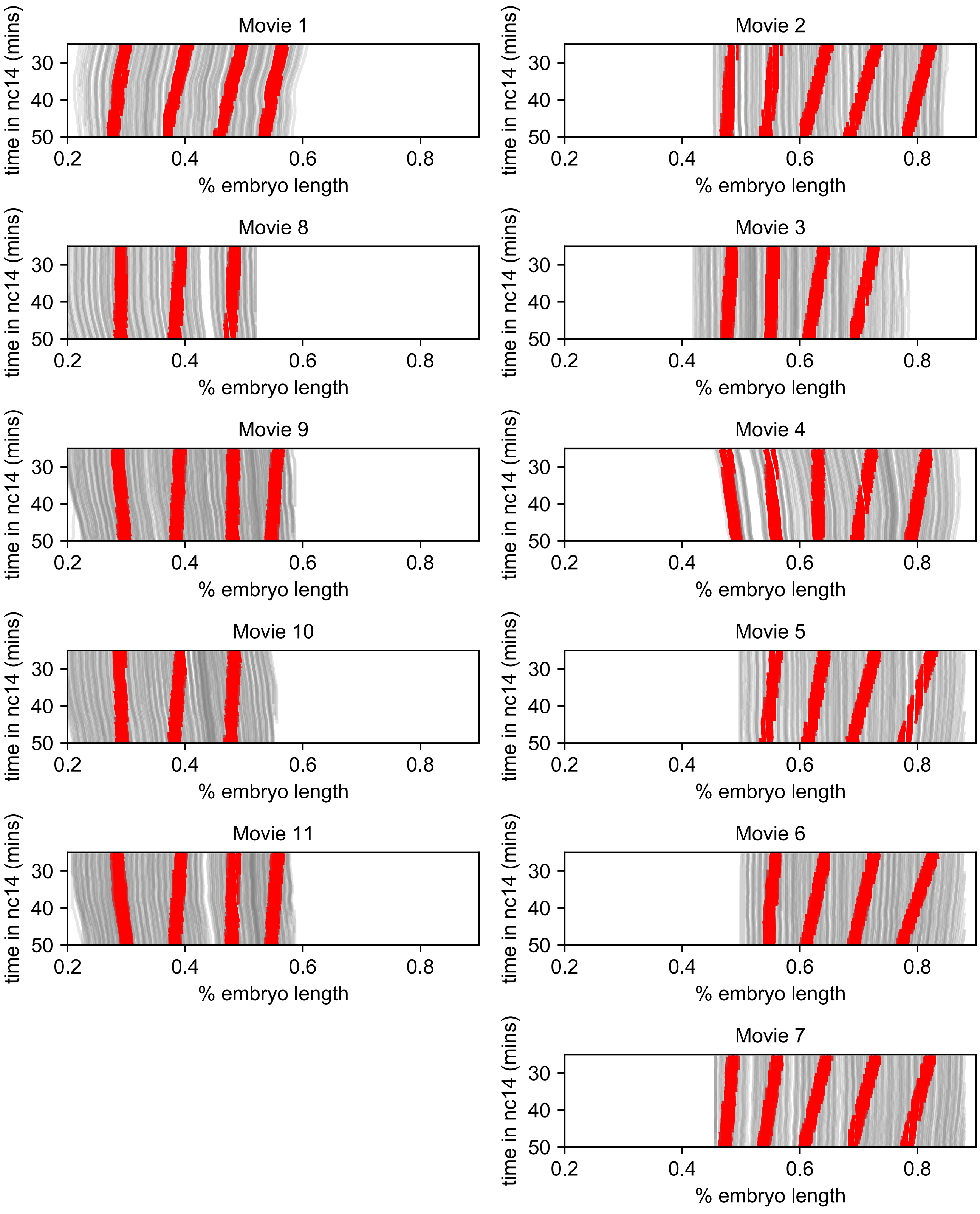

Figure 7-S1. Stripe movement is dominated by the movement of transcriptional activity. Traces of nuclei positions over time (gray lines) from all eleven movies with time points corresponding to transcriptional bursts annotated as in stripes colored red.
