# Supplementary material for: Kinetic sculpting of the seven stripes of the Drosophila even-skipped gene
Source: eLife. 2020 Dec 10;9:e61635. doi: 10.7554/eLife.61635 (PMC7864633; doi:10.7554/eLife.61635)
Supplement: Supplementary file 1. [file elife-61635-supp1.zip › Berrocal_2020_File_S1.tgz!/Berrocal_2020/Figures/Figure1.pdf]

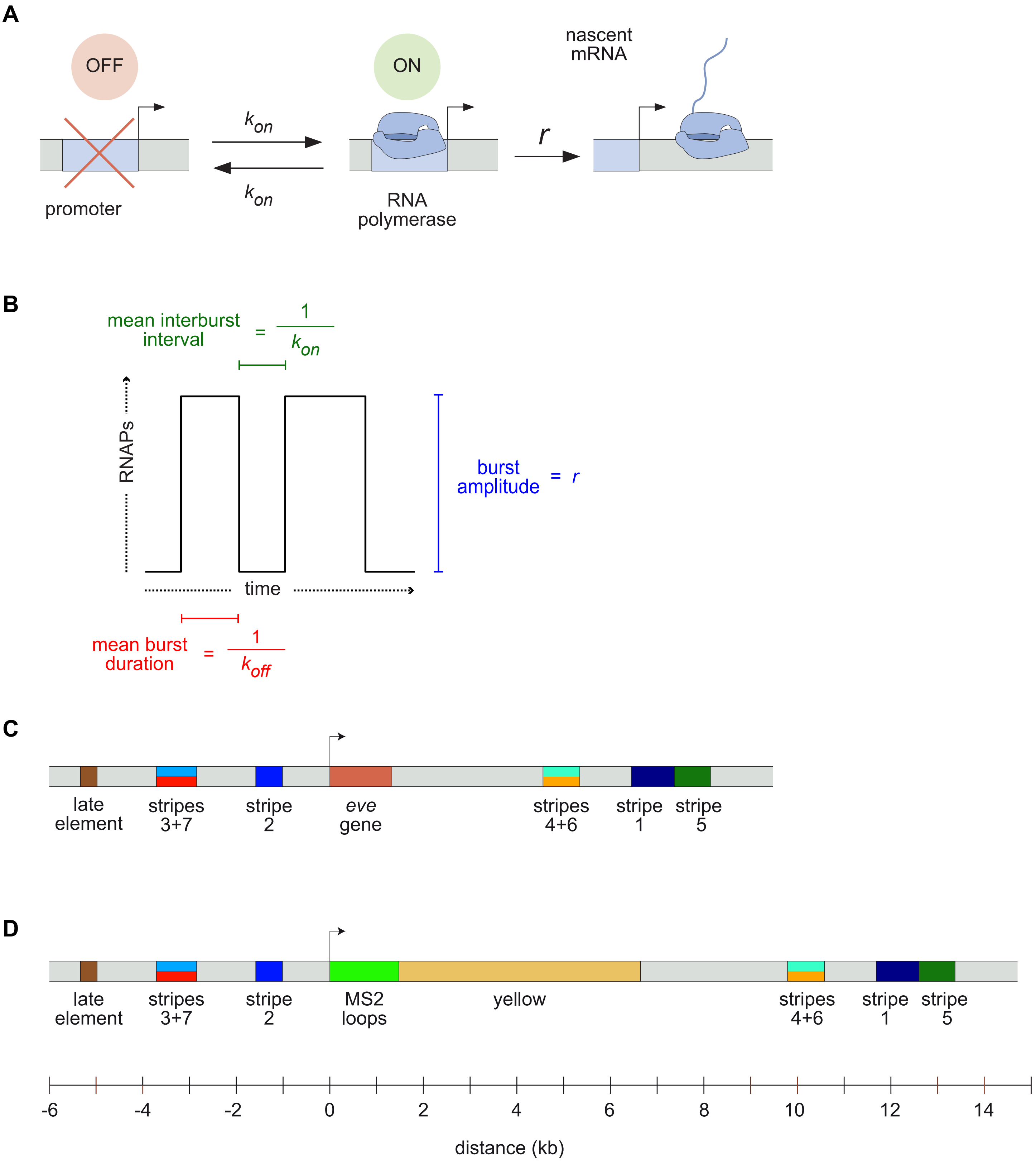

Figure 1. Visualizing live transcription from the seven stripes of *D. melanogaster even-skipped*. (A) Simple model of transcriptional bursting by promoter switching between ON and OFF states. (B) The promoter switching parameters define the burst duration, frequency and amplitude. (C) Wild-type *eve* locus showing the five stripe enhancers (1,2,3+7,4+6,5) and the late enhancer element. Colors for individual stripes are used throughout figures. (D) Layout of the engineered *eve* BAC showing the locations of the MS2 stem loop array and *yellow* gene.
