# Supplementary material for: Kinetic sculpting of the seven stripes of the Drosophila even-skipped gene
Source: eLife. 2020 Dec 10;9:e61635. doi: 10.7554/eLife.61635 (PMC7864633; doi:10.7554/eLife.61635)
Supplement: Supplementary file 1. [file elife-61635-supp1.zip › Berrocal_2020_File_S1.tgz!/Berrocal_2020/Figures/Figure2.pdf]

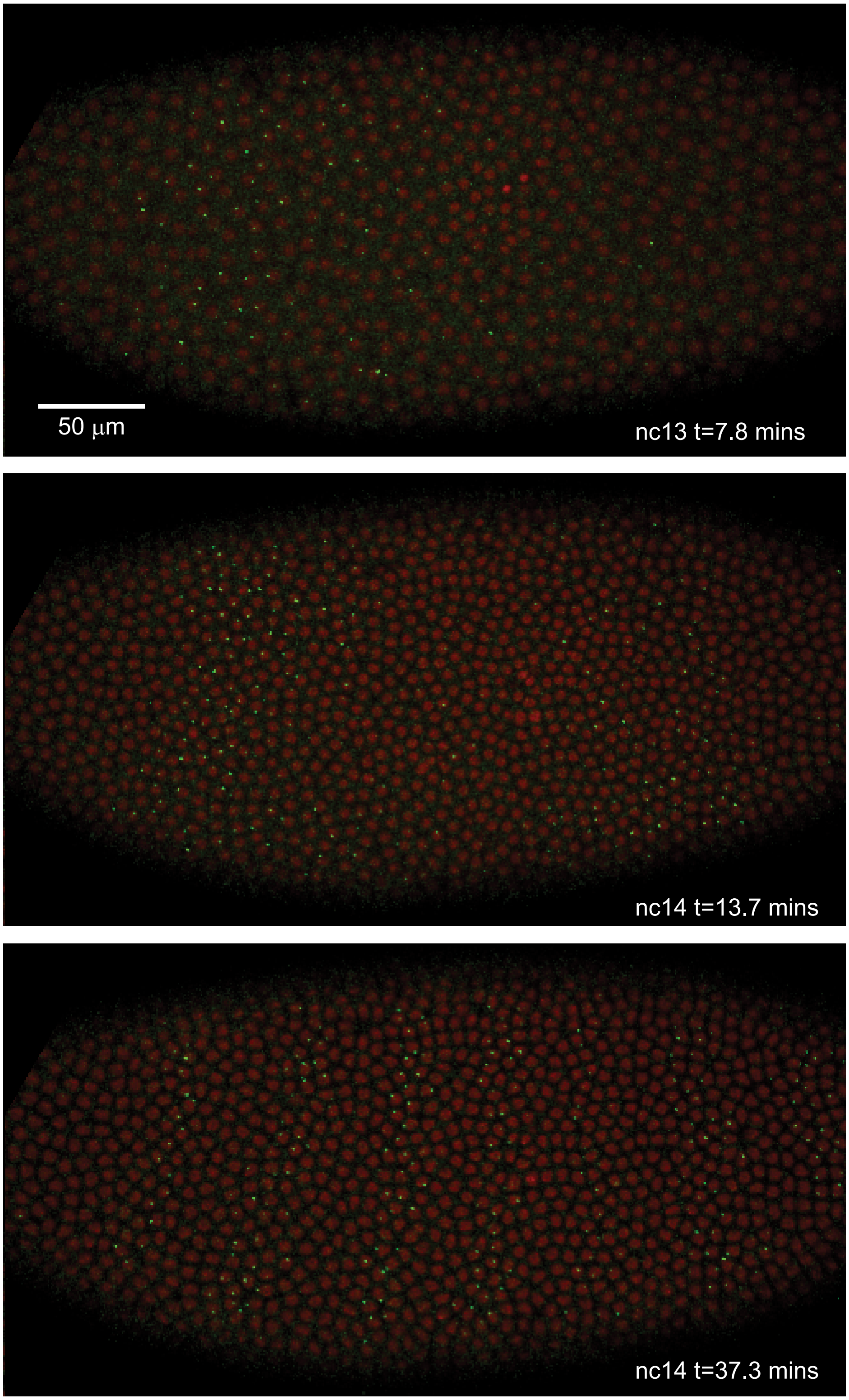

Figure 2. Live expression of even-skipped. Stills from maximum projection renderings of image stacks of an embryo spanning all seven stripes. This movie was collected witha 40x objective for illustration purposes only. Movies used for data analysis were collected at higher resolution as described in the text.
