# Supplementary material for: Kinetic sculpting of the seven stripes of the Drosophila even-skipped gene
Source: eLife. 2020 Dec 10;9:e61635. doi: 10.7554/eLife.61635 (PMC7864633; doi:10.7554/eLife.61635)
Supplement: Supplementary file 1. [file elife-61635-supp1.zip › Berrocal_2020_File_S1.tgz!/Berrocal_2020/Figures/Figure3.pdf]

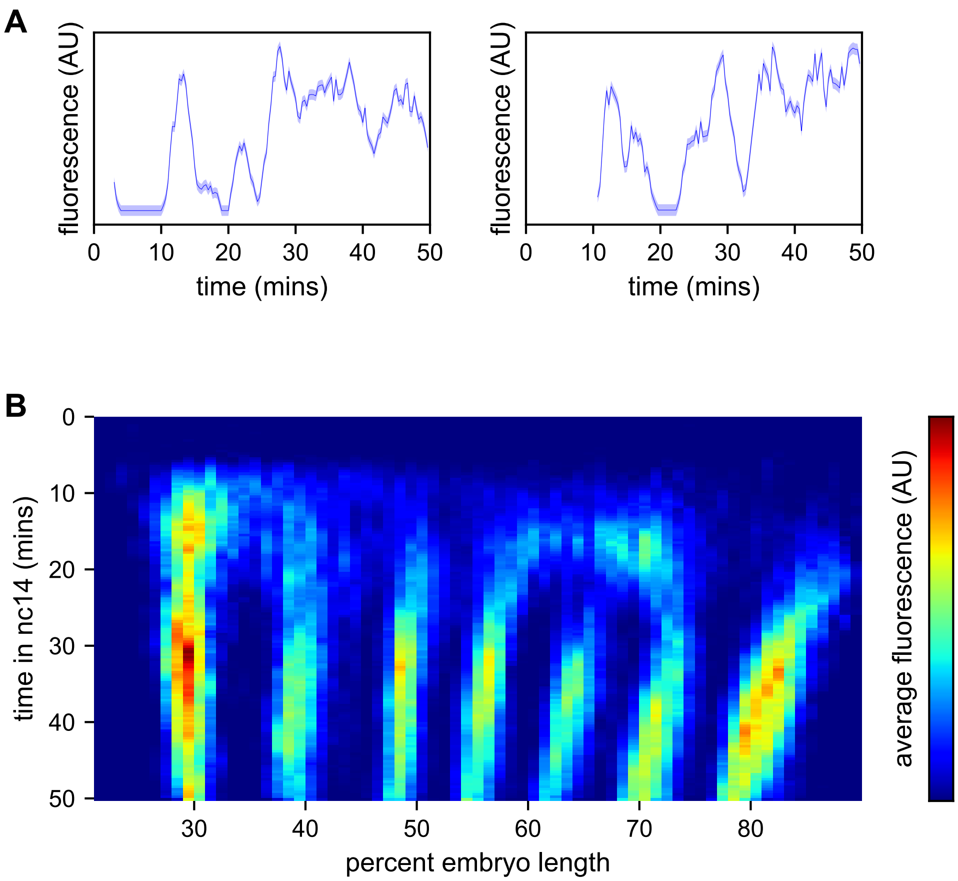

Figure 3. Spatiotemporal dynamics of even-skipped expression. (A) Fluorescence traces from two representative nuclei (particle ID = 1.0163 and 11.0448). (B) Average fluorescence over space and time showing stripe formation, modulation and movement. The time resolution along the y-axis is 20s. The positions of nuclei along the x-axis were registered across movies based on the inferred position of stripe centers, and placed into bins of one percent embryo length, with the average fluorescence of all nuclei in each bin plotted. (A, shading corresponds to the error estimated based on the background fluorescence fluctuations as described in (Garcia et al. 2013)).
