# Supplementary material for: Kinetic sculpting of the seven stripes of the Drosophila even-skipped gene
Source: eLife. 2020 Dec 10;9:e61635. doi: 10.7554/eLife.61635 (PMC7864633; doi:10.7554/eLife.61635)
Supplement: Supplementary file 1. [file elife-61635-supp1.zip › Berrocal_2020_File_S1.tgz!/Berrocal_2020/Figures/Figure7.pdf]

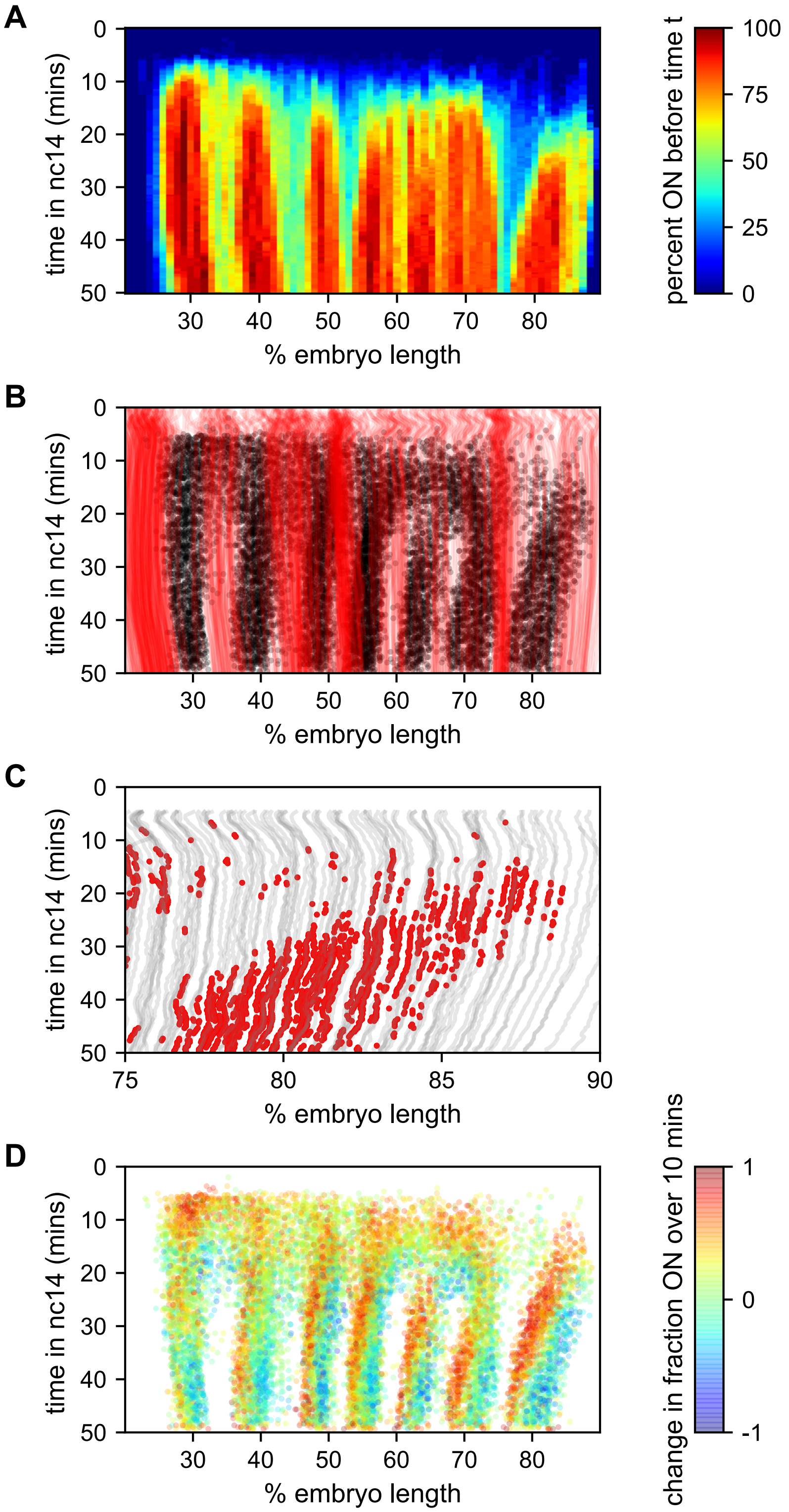

Figure 7. Stripe formation and movement. (A) Fraction of nuclei bursting before time *t* as a function of position along the embryo. (B) Locations of new bursts (black dots) in space and time along with spatiotemporal traces of nuclei that are in the OFF state throughout nc14 (red lines). (C) Traces of nuclei positions over time (gray lines) from stripe 7 region of movie EVE_D6 with timepoints where new bursts initiated colored red to illustrate stripe movement relative to nuclei. (D) Difference in transcriptional activity as defined as the difference between the fraction of the time each nucleus is in the ON state in the subsequent 10 min minus the fraction of time the nucleus is in the ON state in the preceding 10 min. Positive values represent a nucleus turning on or increasing activity, while blue values indicate a nucleus turning off or decreasing activity.
