# Supplementary material for: Kinetic sculpting of the seven stripes of the Drosophila even-skipped gene
Source: eLife. 2020 Dec 10;9:e61635. doi: 10.7554/eLife.61635 (PMC7864633; doi:10.7554/eLife.61635)
Supplement: Supplementary file 1. [file elife-61635-supp1.zip › Berrocal_2020_File_S1.tgz!/Berrocal_2020/Figures/Figure6.pdf]

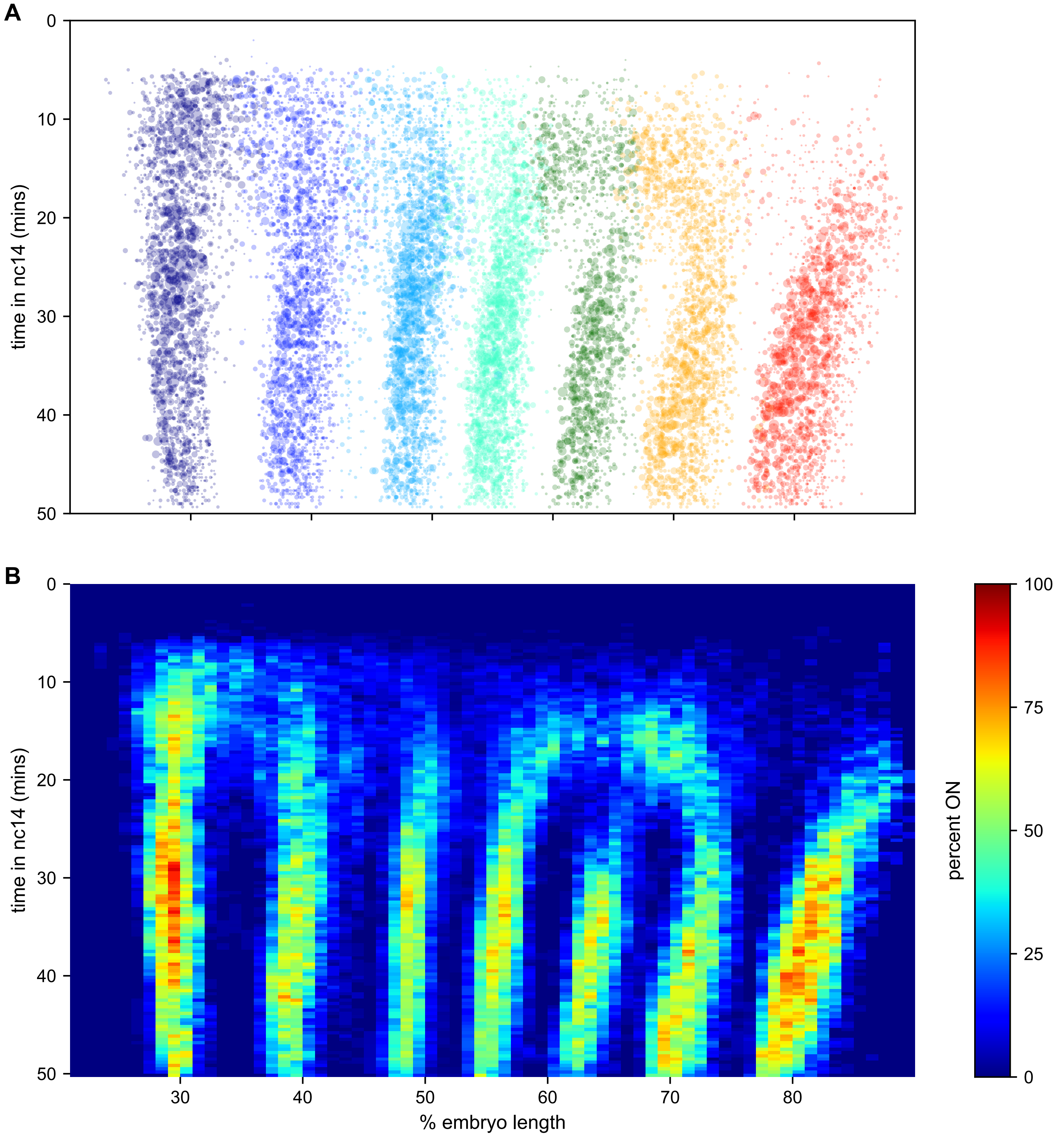

Figure 6. The kinetic fingerprint of *even-skipped* stripe formation. (A) Inferred location of every transcriptional burst in all 11 movies as a function of time and where along the anterio-posterior axis (plotted as fraction of embryo length) each burst occurred. The size of the dot represents the duration of the burst. Collectively the data create a kinetic fingerprint of eve stripe formation. (B) Instantaneous fraction of nuclei in the transcriptionally active ON state as a function of time and position along the embryo.
