# Supplementary material for: Kinetic sculpting of the seven stripes of the Drosophila even-skipped gene
Source: eLife. 2020 Dec 10;9:e61635. doi: 10.7554/eLife.61635 (PMC7864633; doi:10.7554/eLife.61635)
Supplement: Supplementary file 1. [file elife-61635-supp1.zip › Berrocal_2020_File_S1.tgz!/Berrocal_2020/Figures/Figure4.pdf]

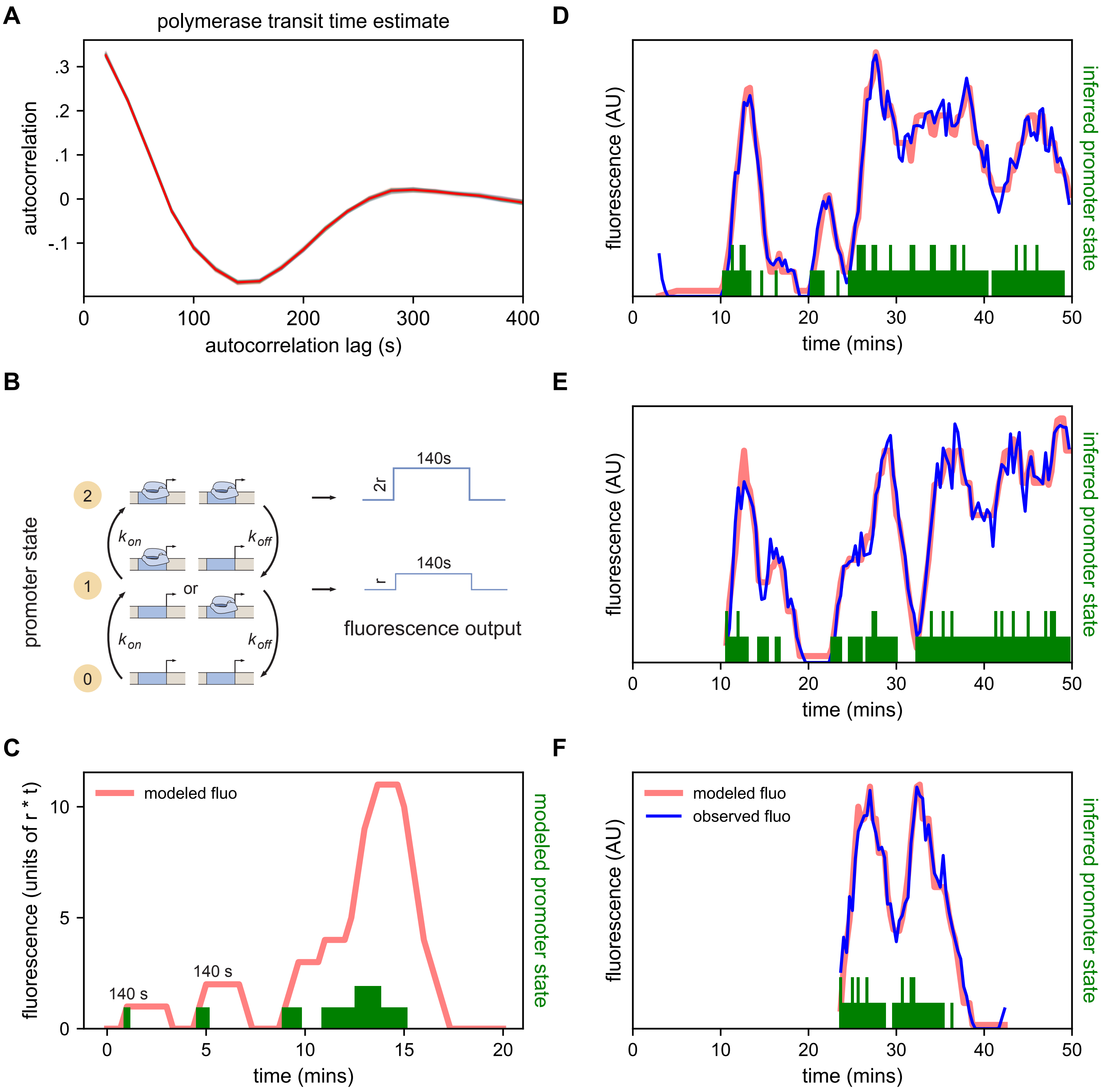

Figure 4. Modeling bursting in individual nuclei. (A) A key parameter in relating fluorescence output to the bursting state of a promoter is the time it takes for a polymerase to transit the gene, which we determined as approximately 140s by examining the autocorrelation (red line) of the change in fluorescence. Gray lines show 100 bootstraps over randomly selected sets of 80% of nuclei; note they almost perfectly overlap the red line. (B) Three state model accounting for post-replication presence of sister chromatids. When either promoter is ON for a short time period Δt, loads polymerases at a constant rate contributing a pulse of polymerase that persists for 140s. (C) Simplified example of the expected observed fluorescence (red line) produced from a hypothetical promoter state sequence. The fluorescence is the sum of the fluorescence pulses produced when one or both promoters are ON (given by the height of the green bars). (D-F) Representative fluorescence traces from individual nuclei (blue lines), inferred bursting pattern (green bars) and fluorescence imputed by cpHMM (red line) for particles 1.0163 (D), 11.0448 (E) and 5.0231 (F).
