# Supplementary material for: Kinetic sculpting of the seven stripes of the Drosophila even-skipped gene
Source: eLife. 2020 Dec 10;9:e61635. doi: 10.7554/eLife.61635 (PMC7864633; doi:10.7554/eLife.61635)
Supplement: Supplementary file 1. [file elife-61635-supp1.zip › Berrocal_2020_File_S1.tgz!/Berrocal_2020/Figures/Figure5.pdf]

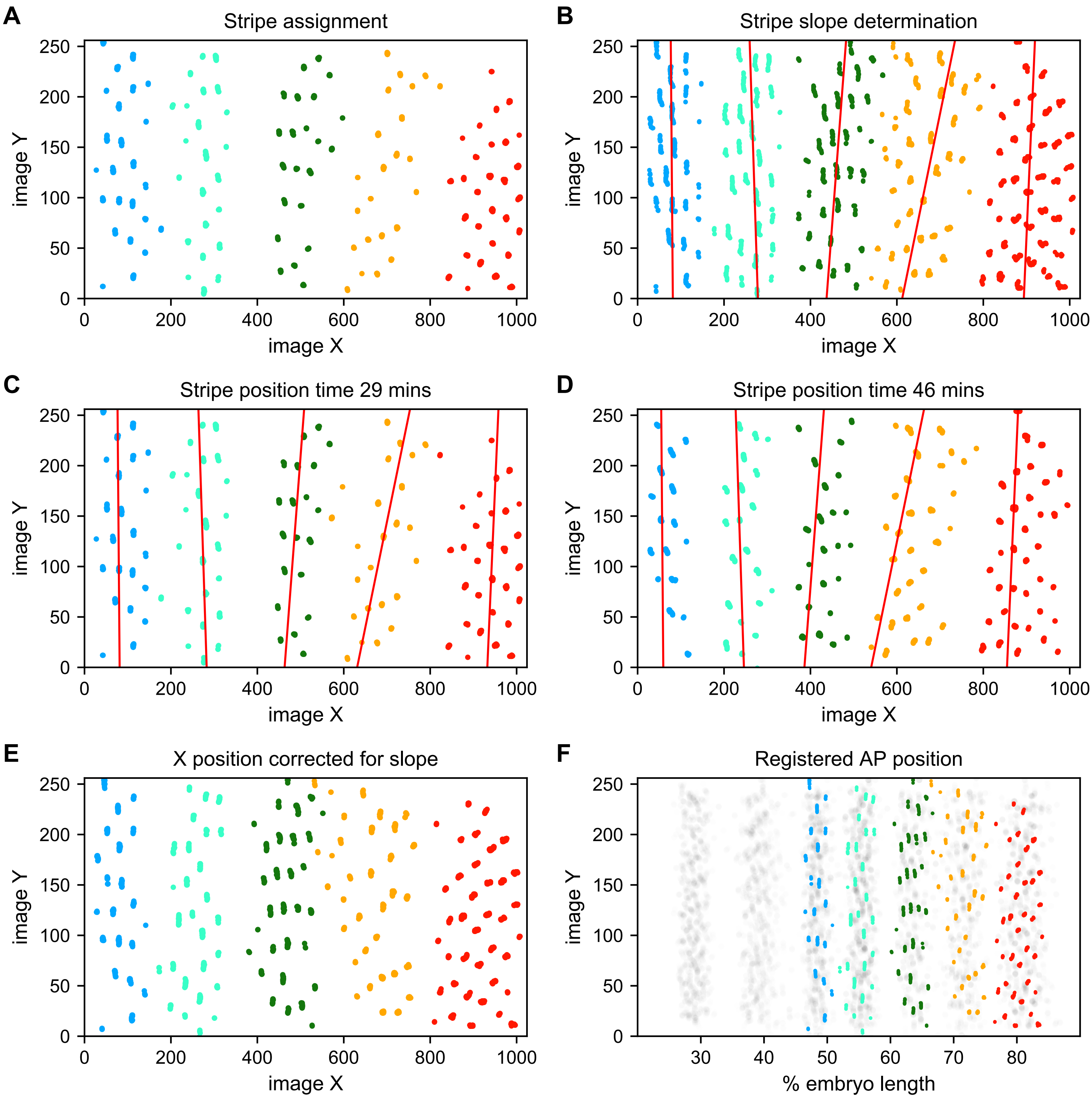

Figure 5. Stripe assignment and alignment. (A) We preliminarily assign bursting nuclei to stripes by applying a Gaussian mixture model to each movie independently in overlapping six-minute time windows with the number of Gaussians equal to the number of stripes captured in that movie. An example is shown here from Movie 2. (B,C,D) We next determine the orientation of each stripe to the imaging axes by fitting a line to coordinates of all nuclei from t > 25 min assigned to that stripe in each movie and time window. (E) We use these fits to generate a linear model of the position of each stripe in each image over time, which makes it possible to reorient the stripe so that it is perpendicular to the image x-axis . (F) The known coordinates of the anterior and posterior poles of the embryo are used to convert the image x-axis to AP position and register the stripes from different movies to each other, as shown here for nuclei from Movie 2 colored by stripe and nuclei corresponding to all other movies drawn in grey.
