# Supplementary material for: Kinetic sculpting of the seven stripes of the Drosophila even-skipped gene
Source: eLife. 2020 Dec 10;9:e61635. doi: 10.7554/eLife.61635 (PMC7864633; doi:10.7554/eLife.61635)
Supplement: Supplementary file 1. [file elife-61635-supp1.zip › Berrocal_2020_File_S1.tgz!/Berrocal_2020/Figures/Images/Figure4_Panels.pdf]

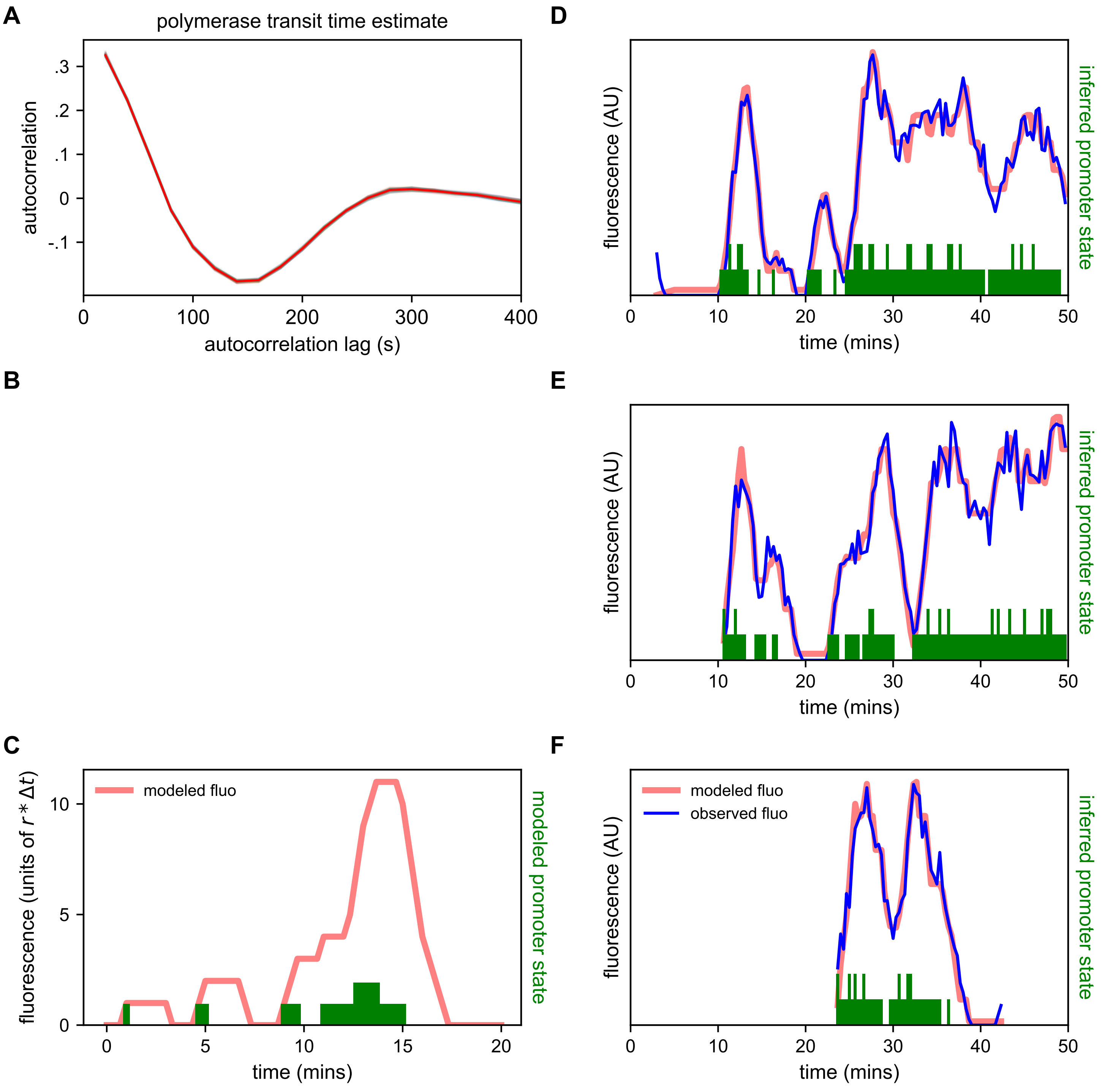

A
polymerase transit time estimate
autocorrelation
.3
.2
.1
0
-.1
0
100
200
300
400
autocorrelation lag (s)
B
C
fluorescence (units of $r * \Delta t$)
modeled fluo
10
5
0
0
5
10
15
20
time (mins)
modeled promoter state
D
fluorescence (AU)
inferred promoter state
time (mins)
E
fluorescence (AU)
inferred promoter state
time (mins)
F
modeled fluo
observed fluo
fluorescence (AU)
inferred promoter state
0
10
20
30
40
50
time (mins)
