# Supplementary material for: Kinetic sculpting of the seven stripes of the Drosophila even-skipped gene
Source: eLife. 2020 Dec 10;9:e61635. doi: 10.7554/eLife.61635 (PMC7864633; doi:10.7554/eLife.61635)
Supplement: Supplementary file 1. [file elife-61635-supp1.zip › Berrocal_2020_File_S1.tgz!/Berrocal_2020/Figures/Images/Figure8_Var_Panels.pdf]

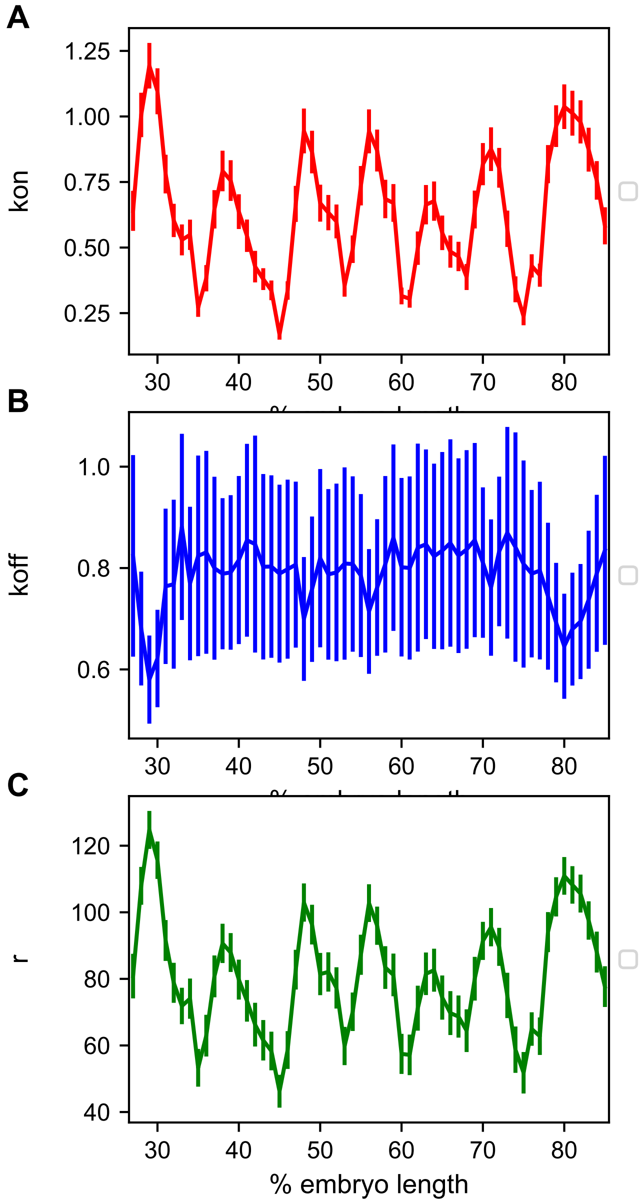
A
kon
1.25
1.00
0.75
0.50
0.25
30
40
50
60
70
80
B
koff
1.0
0.8
0.6
30
40
50
60
70
80
C
r
120
100
80
60
40
30
40
50
60
70
80
% embryo length
