# Supplementary material for: Kinetic sculpting of the seven stripes of the Drosophila even-skipped gene
Source: eLife. 2020 Dec 10;9:e61635. doi: 10.7554/eLife.61635 (PMC7864633; doi:10.7554/eLife.61635)
Supplement: Supplementary file 1. [file elife-61635-supp1.zip › Berrocal_2020_File_S1.tgz!/Berrocal_2020/Figures/Images/Figure5_Panels.pdf]

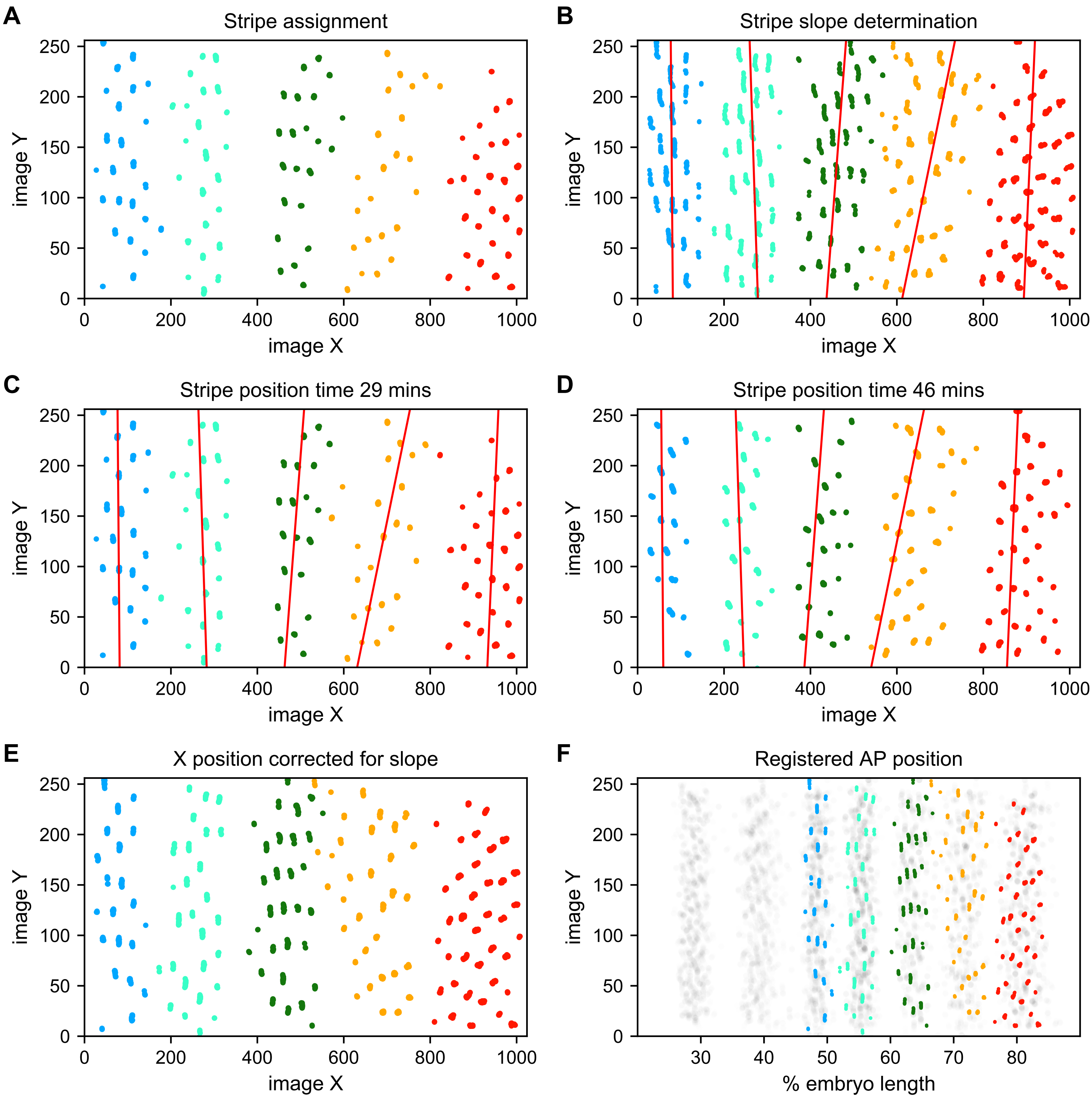
A
Stripe assignment
B
Stripe slope determination
C
Stripe position time 29 mins
D
Stripe position time 46 mins
E
X position corrected for slope
F
Registered AP position
image Y
image X
% embryo length
0
50
100
150
200
250
200
400
600
800
1000
30
40
50
60
70
80
