# Supplementary material for: Kinetic sculpting of the seven stripes of the Drosophila even-skipped gene
Source: eLife. 2020 Dec 10;9:e61635. doi: 10.7554/eLife.61635 (PMC7864633; doi:10.7554/eLife.61635)
Supplement: Supplementary file 1. [file elife-61635-supp1.zip › Berrocal_2020_File_S1.tgz!/Berrocal_2020/Figures/Images/Figure8_Panels.pdf]

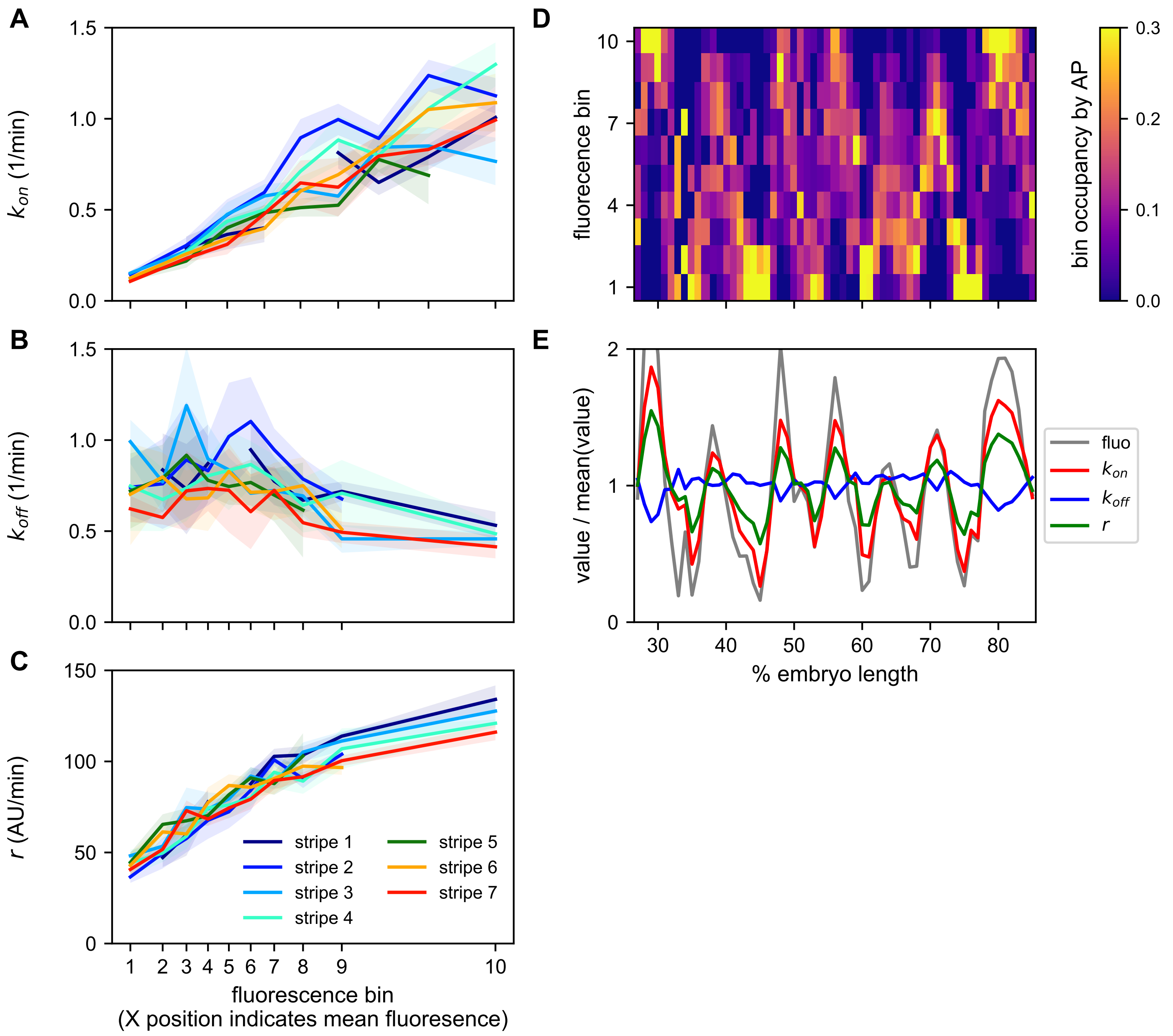
A
B
C
D
E
k_{on} (1/min)
k_{off} (1/min)
r (AU/min)
fluorescence bin
(X position indicates mean fluoresence)
stripe 1
stripe 2
stripe 3
stripe 4
stripe 5
stripe 6
stripe 7
fluorecence bin
bin occupancy by AP
value / mean(value)
% embryo length
fluo
k_{on}
k_{off}
r
