# Supplementary material for: Kinetic sculpting of the seven stripes of the Drosophila even-skipped gene
Source: eLife. 2020 Dec 10;9:e61635. doi: 10.7554/eLife.61635 (PMC7864633; doi:10.7554/eLife.61635)
Supplement: Supplementary file 1. [file elife-61635-supp1.zip › Berrocal_2020_File_S1.tgz!/Berrocal_2020/Figures/Images/Figure7_Panels.pdf]

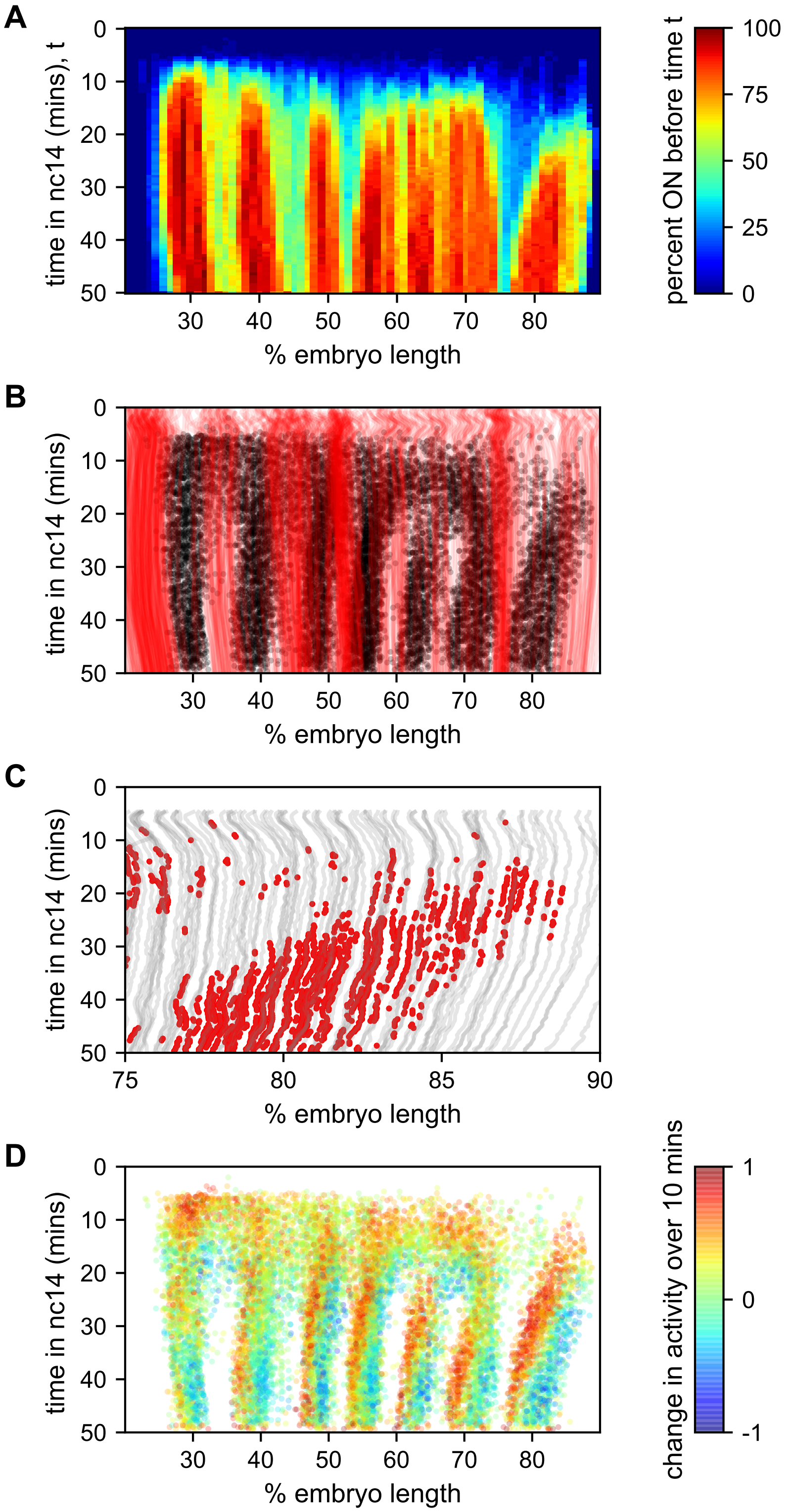
A
time in nc14 (mins), t
0
10
20
30
40
50
30
40
50
60
70
80
% embryo length
percent ON before time t
100
75
50
25
0
B
time in nc14 (mins)
0
10
20
30
40
50
30
40
50
60
70
80
% embryo length
C
time in nc14 (mins)
0
10
20
30
40
50
75
80
85
90
% embryo length
D
time in nc14 (mins)
0
10
20
30
40
50
30
40
50
60
70
80
% embryo length
change in activity over 10 mins
1
0
-1
