# Supplementary material for: Kinetic sculpting of the seven stripes of the Drosophila even-skipped gene
Source: eLife. 2020 Dec 10;9:e61635. doi: 10.7554/eLife.61635 (PMC7864633; doi:10.7554/eLife.61635)
Supplement: Supplementary file 1. [file elife-61635-supp1.zip › Berrocal_2020_File_S1.tgz!/Berrocal_2020/Figures/Images/Figure7-S1_Panels.pdf]

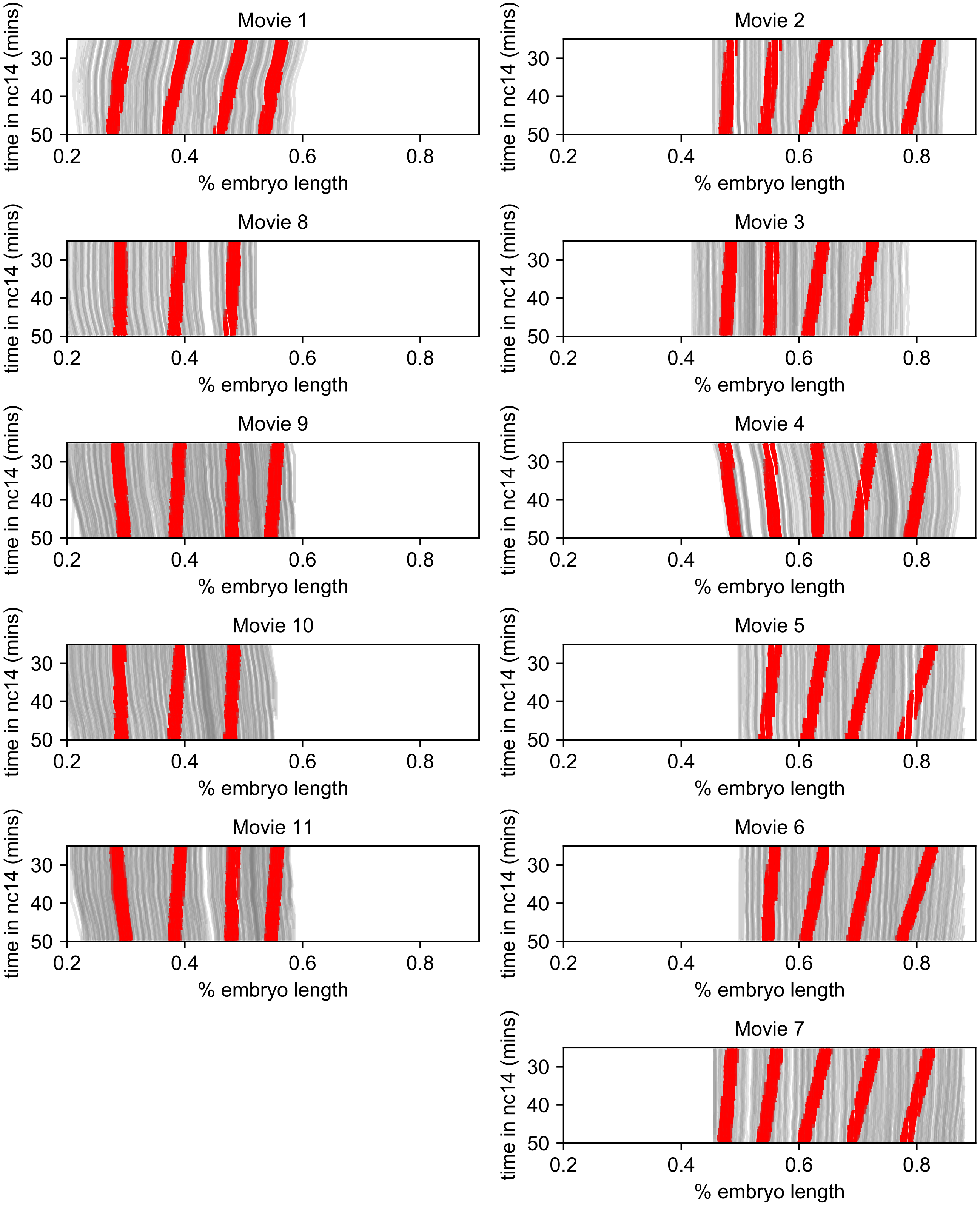
Movie 1
Movie 2
Movie 8
Movie 3
Movie 9
Movie 4
Movie 10
Movie 5
Movie 11
Movie 6
Movie 7
time in nc14 (mins)
% embryo length
30
40
50
0.2
0.4
0.6
0.8
